# Spatial relationship of 2-deoxy-2-[^18^F]-fluoro-D-glucose positron emission tomography and magnetic resonance diffusion imaging metrics in cervical cancer

**DOI:** 10.1186/s13550-018-0403-7

**Published:** 2018-06-15

**Authors:** John M. Floberg, Kathryn J. Fowler, Dominique Fuser, Todd A. DeWees, Farrokh Dehdashti, Barry A. Siegel, Richard L. Wahl, Julie K. Schwarz, Perry W. Grigsby

**Affiliations:** 1 Department of Radiation Oncology, Washington University School of Medicine, 660 S. Euclid Ave, Box 8224, St. Louis, MO 63110 USA; 2 Mallinckrodt Institute of Radiology, Washington University School of Medicine, 660 S. Euclid Ave, Box 8131, St. Louis, MO 63110 USA; 3 Alvin J. Siteman Cancer Center, Washington University School of Medicine, St. Louis, MO USA; 4 Division of Biomedical Statistics and Informatics, Mayo Clinic, 13400 E. Shea Blvd, Scottsdale, AZ 85259 USA; 5 Department of Cell Biology and Physiology, Washington University School of Medicine, St. Louis, MO USA

**Keywords:** PET/MRI, Multimodal imaging, Diffusion imaging, Cervical cancer, Imaging biomarkers

## Abstract

**Background:**

This study investigated the spatial relationship of 2-deoxy-2-[^18^F]-fluoro-D-glucose positron emission tomography ([^18^F]FDG-PET) standardized uptake values (SUVs) and apparent diffusion coefficients (ADCs) derived from magnetic resonance (MR) diffusion imaging on a voxel level using simultaneously acquired PET/MR data.

We performed an institutional retrospective analysis of patients with newly diagnosed cervical cancer who received a pre-treatment simultaneously acquired [^18^F]FDG-PET/MR. Voxel SUV and ADC values, and global tumor metrics including maximum SUV (SUV_max_), mean ADC (ADC_mean_), and mean tumor-to-muscle ADC ratio (ADC_T/M_) were compared. The impacts of histology, grade, and tumor volume on the voxel SUV to ADC relationship were also evaluated. The potential prognostic value of the voxel SUV/ADC relationship was evaluated in an exploratory analysis using Kaplan-Meier/log-rank and univariate Cox analysis.

**Results:**

Seventeen patients with PET/MR scans were identified. There was a significant inverse correlation between SUV_max_ and ADC_mean_, and SUV_max_ and ADC_T/M_. In the voxelwise analysis, squamous cell carcinomas (SCCAs) and poorly differentiated tumors showed a consistent significant inverse correlation between voxel SUV and ADC values; adenocarcinomas (AdenoCAs) and well/moderately differentiated tumors did not. The strength of the voxel SUV/ADC correlation varied with metabolic tumor volume (MTV). On log-rank analysis, the correlation between voxel SUV/ADC values was prognostic of disease-free survival (DFS).

**Conclusions:**

In this hypothesis-generating study, a consistent inverse correlation between voxel SUV and ADC values was seen in SCCAs and poorly differentiated tumors. On univariate statistical analysis, correlation between voxel SUV and ADC values was prognostic for DFS.

**Electronic supplementary material:**

The online version of this article (10.1186/s13550-018-0403-7) contains supplementary material, which is available to authorized users.

## Background

Despite advances in both prevention of cervical cancer with HPV vaccination and screening for pre-malignant and early stage disease using the Papnicolaou test, deaths from cervical cancer have remained steady in the USA since the mid-2000s [1]. Worldwide, cervical cancer is the fourth leading cause of cancer deaths [2]. A number of prognostic imaging markers have been developed in cervical cancer that can identify patients at high risk for disease progression, recurrence, and death. Imaging markers derived from 2-deoxy-2-[^18^F]-fluoro-D-glucose positron emission tomography ([^18^F]FDG-PET), such as the maximum standardized uptake value (SUV_max_), are the most studied and best established [3–6]. More recently, magnetic resonance imaging (MRI)-derived markers, particularly diffusion metrics, such as the apparent diffusion coefficient of water (ADC), have been studied as additional prognostic imaging markers for cervical cancer [7–11].

To date, tumor response on post-treatment [^18^F]FDG-PET imaging has been shown to be one of the most powerful prognostic imaging markers in cervical cancer [12]. Although this is useful as a prognostic tool, it cannot be used to guide treatment decisions prospectively. A more robust pre-therapy marker would therefore be valuable. Greater understanding of the underlying biology of tumors at high risk for recurrence is also needed, and multimodal imaging markers, for example, utilizing both [^18^F]FDG-PET and MRI data, might provide insight into the physiologic and biologic properties of aggressive tumors.

The spatial relationship between [^18^F]FDG-PET metrics and MRI-derived diffusion metrics, namely ADC values, is not yet clear. The relationship of whole tumor imaging metrics, such as SUV_max_ and minimum ADC (ADC_min_), has previously been studied in cervical cancer as well as several other cancers, with a suggestion that SUV and ADC metrics are inversely correlated [13–20]. Previous work has also shown that qualitatively, regions of intense [^18^F]FDG uptake within primary cervical tumors correlate with regions with lower ADC values [21]. Simultaneously acquired PET/MRI allows for comparison of PET- and MRI-derived imaging markers on a fine spatial scale, for example, on the voxel level if the matrices of PET and MRI images are made the same size. Such a comparison would provide better understanding of the relationship between these imaging markers. The relationship between these metrics in cancers with different histologies, namely squamous cell carcinoma (SCCA) and adenocarcinoma (AdenoCA), and grades will also be important to establish as prior work has shown that histology and differentiation affect [^18^F]FDG uptake [22]. The relationship between SUV and ADC may also yield a more robust prognostic imaging marker than can be obtained with either modality alone.

The primary purpose of this study was to compare pre-treatment [^18^F]FDG-PET SUV and MRI ADC values on a voxel-by-voxel basis in primary cervical cancer tumors in patients with pre-treatment simultaneous PET/MR scans. Global metrics, such as the SUV_max_ and the mean ADC (ADC_mean_), were also compared, and the relationship between SUV and ADC values was compared between different histologies and different grade tumors. The impact of tumor volume on the voxel SUV to ADC relationship was also evaluated. Finally, the ability of SUV and ADC metrics, and the strength of the correlation between voxel SUV and ADC, to predict disease-free survival (DFS) was investigated as an exploratory analysis.

## Methods

### Patients

This retrospective study using existing imaging data and medical records was conducted with institutional review board approval with a waiver of consent. Some of the patients included in this study were enrolled on prospective imaging trials for which they provided written consent; the results reported here are not related to the aims of those studies. Patients from our institution with biopsy proven cervical cancer with pre-treatment simultaneously acquired PET/MR examinations obtained between October 2011 and June 2016 were identified. This included patients subsequently treated with definitive chemoradiation and patients subsequently treated with surgery. Patient clinical and demographic characteristics were obtained through review of the medical record. Patient outcome data, namely disease status, time to recurrence, and time to death, were obtained through review of the medical record and publicly available records.

### Patient preparation and image acquisition

Patients fasted for at least 4 h, and blood glucose was required to be less than 11.1 mmol/L prior to [^18^F]FDG injection. The radiotracer was administered intravenously with a mean activity of 512.82 MBq, 6.80 MBq/kg (range 218.3–710.4 MBq, 2.91–9.07 MBq/kg). The majority of patients (10/17, 58.8%) had Foley catheters placed prior to radiotracer injection. Eleven patients included in this study were also enrolled on a PET/MRI optimization study, in which they first underwent a PET/CT according to our standard institutional protocol, immediately followed by a PET/MRI. In these patients, the average time from the radiotracer injection to the start of the PET/MR acquisition was 135 min (range 103–171 min).

For PET/MRI scanning, all imaging was performed on a Siemens Biograph mMR integrated PET/MRI scanner (Siemens Medical Solutions USA, Inc.). Whole-body static PET images were acquired at multiple bed positions for 3–4 min per position. Images were reconstructed using an ordinary Poisson-ordered subset expectation-maximization algorithm (OP-OSEM) to a voxel size of 4.17 × 4.17 × 2.03 mm. Attenuation correction was performed using the MRI data, and scatter correction was performed using the system’s model-based algorithm.

MR images were acquired simultaneously with the PET data. MR sequences included a Dixon sequence for PET attenuation correction, a half-Fourier acquired single-shot turbo spin-echo (HASTE) sequence for localization and anatomical overview, a T2 high-resolution isotropic fast spin-echo sequence (SPACE) to characterize lesion morphology, and a contrast-enhanced dynamic 3D T1 fat suppression sequence. Diffusion-weighted images were acquired using an echo-planar diffusion imaging sequence with an axial acquisition, slice thickness of 5 mm, in plane resolution of 3.4 mm, and *b* values of either 50–500–1000, 50–500–800, or 50–400–600. ADC maps were produced by the scanner from the diffusion-weighted images.

### Image analysis

All image analysis was performed on a MIM Vista workstation (MIM Software Inc.). [^18^F]FDG-PET images were resampled to the ADC image matrix, such that the voxels between the modalities matched. We focused our analysis on regions with diffusion restriction and high [^18^F]FDG avidity. Therefore, gross tumor volumes of interest (VOIs) were manually defined on the ADC images by one of the authors (JMF) to encompass the hypointense, diffusion-restricted region of the tumor, using the T2 and [^18^F]FDG-PET images to help verify the location of the tumor, according to our institutional practice [23, 24]. In cases where there was no discernable hypointense tumor on the ADC image, PET images were segmented to define the tumor based on a threshold equal to 40% of the SUV_max_ [25]; this was the case in three of the AdenoCAs. Global tumor PET imaging metrics obtained included the SUV_max_, metabolic tumor volume (MTV) defined with a 40% of the SUV_max_ threshold, mean SUV (SUV_mean_) determined within the MTV, total lesion glycolysis (TLG) obtained by multiplying the MTV by the SUV_mean_, and the SUV_mean_ normalized by the average SUV of adjacent gluteal muscle (SUV_T/M_). Global tumor MRI metrics included the ADC tumor volume (ADC_vol_), ADC_min_, mean ADC (ADC_mean_), and the ratio of the mean tumor ADC to the mean ADC of the adjacent gluteal muscle (ADC_T/M_).

Primary tumor SUV_max_, SUV_mean_, and SUV_T/M_ were compared to the ADC_mean_, ADC_T/M_, and ADC_min_ of the tumor. SUV and ADC values were also compared on a voxel-by-voxel basis within each tumor. The global tumor metrics outlined above and voxel-by-voxel correlation between SUV and ADC were also compared between SCCAs and AdenoCAs. Finally, the impact on tumor grade (poorly vs. well/moderately differentiated) and tumor volume (using MTV) on the relationship of voxel SUV and ADC values was evaluated. The author performing the image analysis (JMF) was blinded to patient outcomes at the time of image analysis.

### Statistics

Correlation between global tumor SUV and ADC values (e.g., SUV_max_ and ADC_mean_, SUV_max_, and ADC_T/M_), and between voxel SUV and ADC values, was determined using the Pearson correlation coefficient. The correlation between the voxel SUV/ADC relationship and MTV was also determined using the Pearson correlation coefficient. The statistical significance of the correlations was determined using two-sided *t* statistics. Imaging metrics from SCCAs and AdenoCAs, and poorly and well/moderately differentiated tumors, were compared using the two-sided Mann-Whitney *U* test. The prognostic value of the correlation between voxel SUV and ADC values (using Pearson’s *r*), as well as the SUV_max_, MTV, ADC_mean_, and ADC_T/M_ were determined using Kaplan-Meier and log-rank analysis, and Cox univariate proportional hazards analysis for disease-free survival (DFS). This analysis was performed using SPSS (IBM Analytics). Variables were treated as categorical for both the Cox proportional hazards and the log-rank analysis, dichotomized by the median for each imaging metric.

## Results

### Patient characteristics

A total of 17 patients were identified, 9 (52.9%) with SCCA, 6 (35.3%) with AdenoCA, and 1 (5.9%) each of small cell carcinoma and adenosquamous carcinoma. The majority of patients (14, 82.4%) were treated with definitive chemoradiation therapy. Median follow-up for the cohort was 23.9 months. The 2 year DFS and OS were 76.5 and 82.4%, respectively. Patient characteristics are summarized in Table 1.

**Table 1:** Characteristics of the patient cohort

Characteristic	No. of patients (%)
Histology	
SCCA	9 (52.9%)
AdenoCa	6 (35.3%)
Small cell	1 (5.9%)
Adenosquamous	1 (5.9%)
Grade	
Well differentiated	3 (17.6%)
Moderately differentiated	3 (17.6%)
Poorly differentiated	11 (64.7%)
Treatment modality	
Surgery only	3 (17.6%)
Chemo-RT	14 (82.4%)
FIGO stage	
IA	0 (0%)
IB1	4 (23.5%)
IB2	7 (41.2%)
IIA	1 (5.9%)
IIB	0 (0%)
IIIA	1 (5.9%)
IIIB	3 (17.6%)
IVA	0 (0%)
IVB	1 (5.9%)
Nodal involvement	
None	7 (41.2%)
Pelvic	7 (41.2%)
Para-aortic	2 (11.8%)
Supraclavicular	1 (5.9%)

### Global tumor metrics

Global tumor imaging metrics assessed included the PET metrics of SUV_max_, MTV, and TLG, and the MRI diffusion metrics of ADC_min,_ ADC_mean_, ADC_T/M_, and ADC_vol_. The average values of these metrics were compared between SCCA and AdenoCA tumors (Table 2). There was not a statistically significant difference between any of these values between SCCAs and AdenoCAs in this patient cohort.

**Table 2:** Comparison of global tumor imaging metrics between SCCAs and AdenoCAs

	SCCAs	AdenoCAs	*p* value
ADC_mean_	958.9	1198.5	0.18
ADC_T/M_	0.77	0.95	0.11
ADC_min_	402.6	331.5	0.27
SUV_max_	13.2	11.1	0.86
MTV	41.12	24.7	0.33
TLG	280.9	258.9	0.61
ADC_vol_	47.16	49.93	0.61

**Fig. 1:**
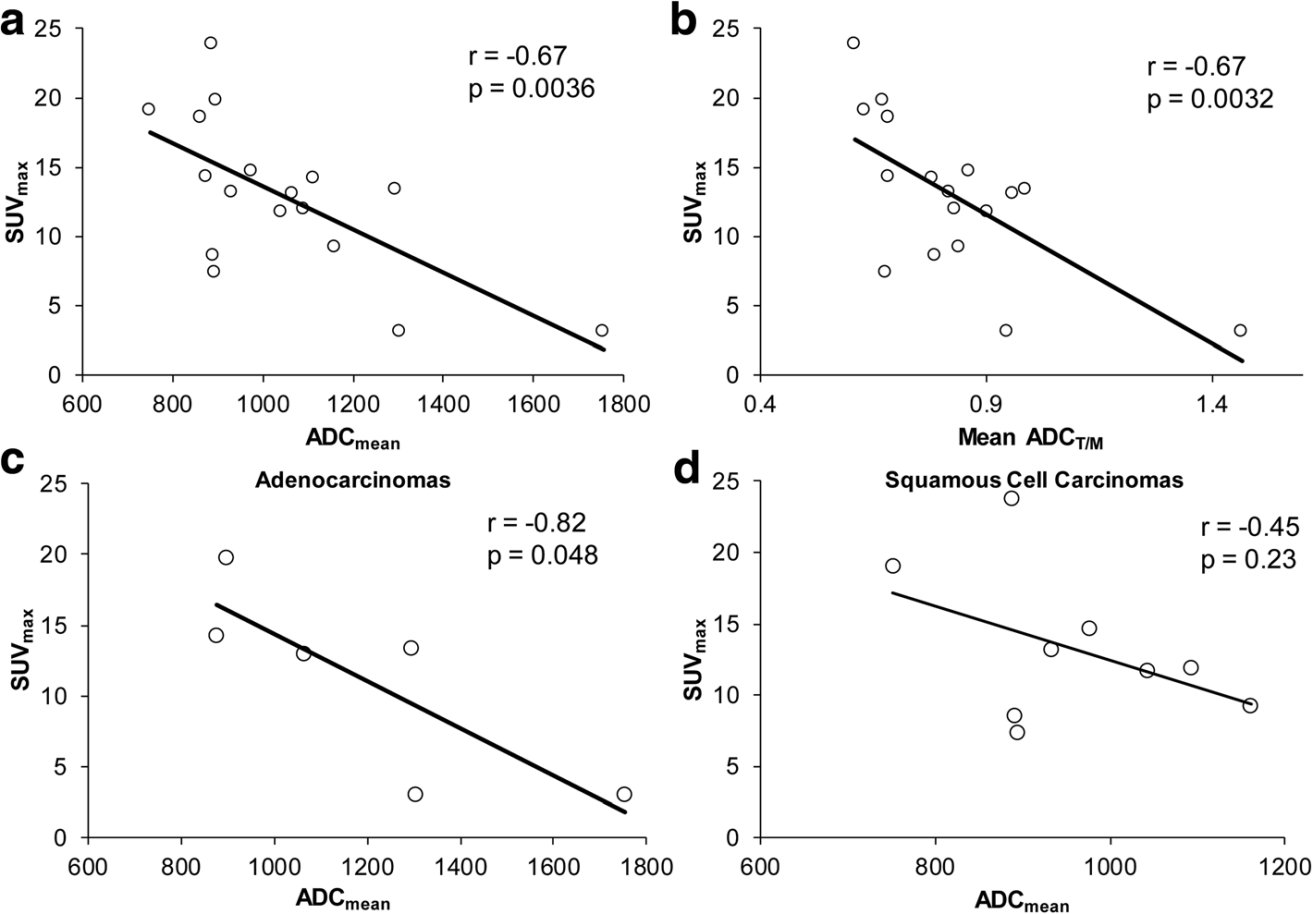
Comparison of the global tumor metrics of SUV_max_ and ADC_mean_ (**a**) and SUV_max_ and ADC_T/M_ (**b**) for all tumors. When SCCAs and AdenoCAs are considered separately, there is still a significant inverse correlation between SUV_max_ and ADC_mean_ for AdenoCAs (**c**), and though there is an inverse correlation in SCCAs, it does not meet significance (**d**)

The correlation between SUV_max_ and ADC_mean_, and SUV_max_ and ADC_T/M_ were evaluated for all tumors (Fig. 1a, b). There was a significant inverse correlation between SUV_max_ and both of these ADC metrics. The correlation between SUV_max_ and ADC_min_ was also evaluated and was non-significant (*r* = − 0.43, *p* = 0.08). There was also a significant correlation between SUV_mean_ and ADC_mean_ (*p* = 0.007) and between SUV_mean_ and ADC_T/M_ (*p* = 0.008). There was no significant correlation between SUV_T/M_ and either ADC_mean_ (*p* = 0.08) or ADC_T/M_ (*p* = 0.06) (Additional file 1: Figure S1). The correlation between SUV_max_ and ADC_mean_ was also evaluated for SCCAs and AdenoCAs. There was an inverse correlation between these metrics for both histologies, which remained significant in AdenoCAs but did not meet the threshold for significance in SCCAs (Fig. 1c, d). The inverse correlation between SUV_mean_ and ADC_mean_, and SUV_mean_ and ADC_T/M_, did not maintain significance when dividing tumors into AdenoCAs (*p* = 0.07 and 0.09, respectively) and SCCAs (*p* = 0.15 and 0.21, respectively).

### Voxelwise comparison

**Fig. 2:**
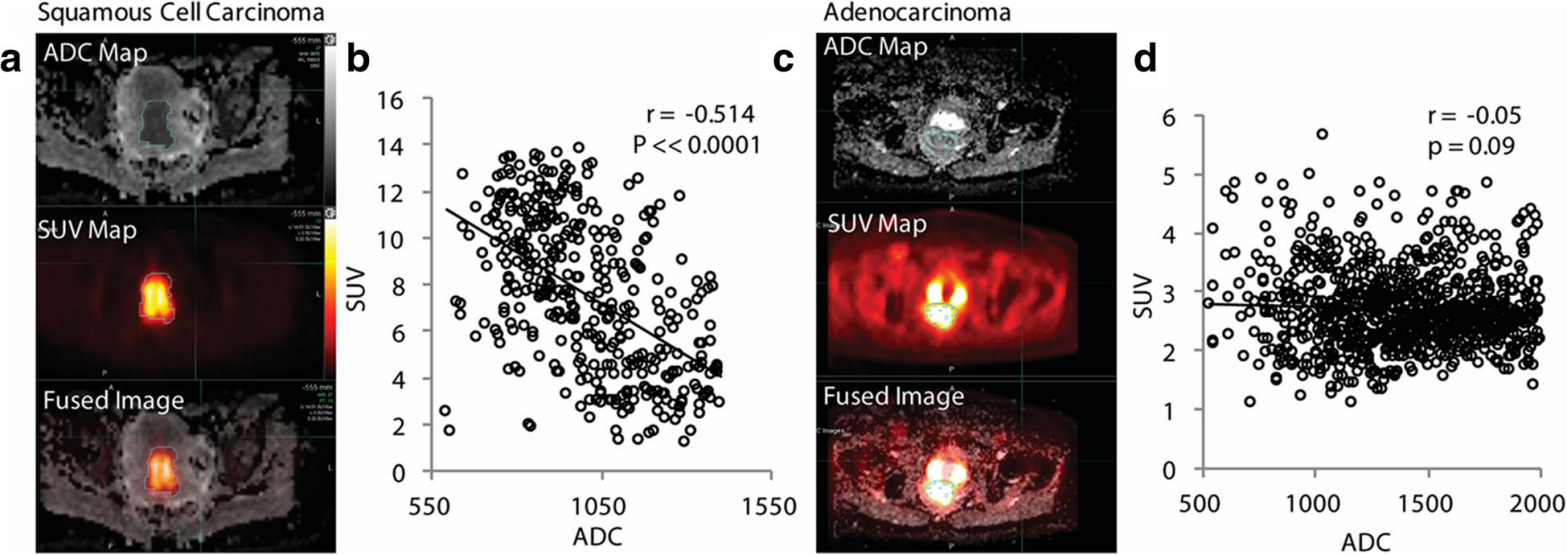
Representative SCCA tumor (**a**) and the corresponding plot comparing the ADC and SUV of each voxel from the primary tumor (**b**). A representative AdenoCA tumor (**c**) and its corresponding plot comparing ADC and SUV (**d**) are also shown. Most SCCA tumors showed a correlation between voxel ADC and SUV values, whereas most of the AdenoCAs did not

**Fig. 3:**
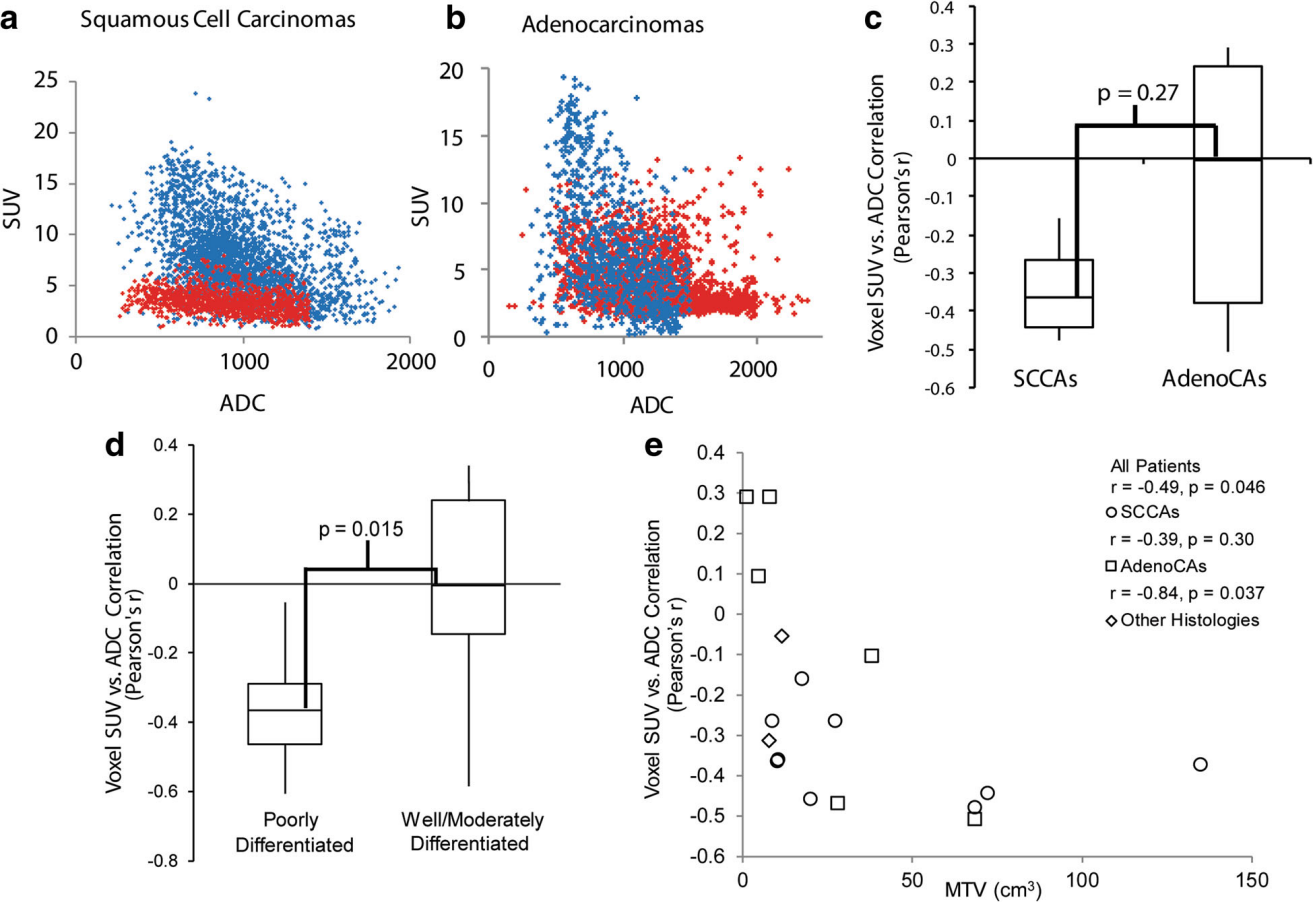
The voxel ADC vs. SUV comparisons for all SCCA tumors (**a**) and all AdenoCA tumors (**b**). Each point represents a single voxel, and all voxels from all tumors are displayed together. For the SCCAs (**a**), in the majority of tumors, there is a steeper SUV vs. ADC slope (blue); for two tumors, the slope is not as steep, but there is still a correlation (red). For the AdenoCAs (**b**), one tumor has a steep SUV vs. ADC slope (blue), but voxels from the rest of the tumors are more erratically scattered (red). When the correlation between ADC and SUV, as measured by Pearson’s *r*, is compared between SCCAs and AdenoCAs, there is a consistent inverse correlation between these values in SCCAs, but not AdenoCAs (**c**). Poorly differentiated tumors have a significantly greater inverse correlation between voxel SUV and ADC compared to well/moderately differentiated tumors (**d**). The correlation between voxel SUV and ADC varies with MTV, though this relationship is primarily driven by the AdenoCAs (**e**)

Individual voxel SUV and ADC values were compared for each primary tumor. A linear least-squares fit was performed for each tumor, and the slope and Pearson’s correlation coefficients were determined. Example patients with SCCA and AdenoCA tumors and the voxel comparisons of ADC and SUV are shown in Fig. 2. SCCA tumors appeared dark on the ADC images, corresponding to areas of intense [^18^F]FDG uptake, and voxel-by-voxel comparison of ADC and SUV showed an inverse correlation in this example (Fig. 2a, b). In contrast, while some AdenoCAs showed this correlation, others appeared heterogeneous on the ADC images, and in the example patient, voxel-by-voxel comparison of ADC and SUV revealed little correlation (Fig. 2c, d). When all patients with SCCAs were grouped together, all but two patients showed a strong inverse relationship between ADCs and SUVs; the two outliers still had a significant correlation between SUV and ADC but with a less steep slope (Fig. 3a). When all AdenoCAs were grouped together, one tumor showed a strong inverse relationship between ADC and SUV, but other tumors showed little to no correlation (Fig. 3b).

We then compared the Pearson correlation coefficients of the SCCAs and AdenoCAs. The adenosquamous cancer and small cell cancer were therefore excluded from this analysis. All of the SCCAs showed a statistically significant inverse correlation between SUV and ADC, with a mean Pearson’s *r* = − 0.35. In contrast, though there were three AdenoCAs with a statistically significant inverse correlation between SUV and ADC, there was a wide spread of Pearson’s correlation coefficients, with a mean *r* = − 0.068. The difference in the mean Pearson’s correlation coefficients between SCCAs and AdenoCAs did not meet statistical significance in this small patient cohort with a broad range of correlation coefficients in the AdenoCAs (*p* = 0.27, Fig. 3c). We also compared the Pearson correlation coefficients between poorly and well/moderately differentiated tumors. All but one poorly differentiated tumor showed a statistically significant inverse correlation between voxel SUV and ADC with a mean *r* = − 0.36, and 4/6 well/moderately differentiated tumors had a statistically significant inverse correlation between voxel SUV and ADC with a mean *r* = − 0.006 (Fig. 3d). The difference in the mean Pearson correlation coefficient between poorly and well/moderately differentiated tumors did reach statistical significance (*p* = 0.015). Notably, 4/6 of the AdenoCAs were also well/moderately differentiated, and 7/9 of the SCCAs were poorly differentiated.

Finally, we investigated the impact of tumor volume on the voxel SUV/ADC correlation. When all tumors are considered, there is a significant inverse correlation between the voxel SUV/ADC relationship and MTV (Fig. 3e, *r* = − 0.49, *p* = 0.046). However, three of the small tumors with no inverse correlation between voxel SUV and ADC were AdenoCAs. Considering SCCAs and AdenoCAs separately, there is still a significant inverse correlation between MTV and the voxel SUV/ADC relationship in AdenoCAs (*r* = − 0.84, *p* = 0.04), but not in SCCAs (*r* = − 0.39, *p* = 0.30).

### PET and diffusion MR imaging metrics and patient outcomes

**Fig. 4:**
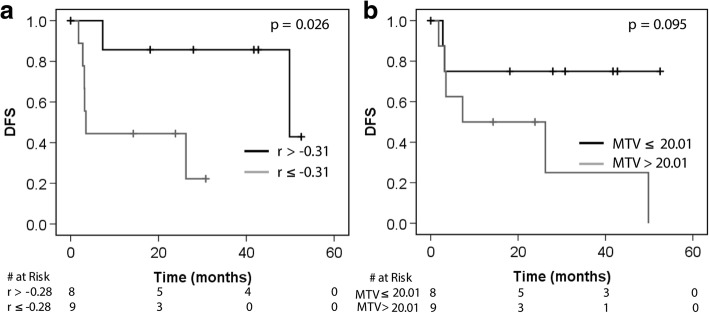
Kaplan-Meier curves for DFS with patients stratified by the median Pearson’s *r* for the correlation between voxel ADC and SUV (**a**) and the median MTV (**b**). Tumors with a stronger inverse correlation between ADC and SUV show poorer disease outcomes

Univariate statistical analysis of the prognostic value of the correlation between voxel SUV and ADC values was performed as an exploratory analysis. The prognostic value of established PET and MRI imaging metrics was also evaluated in this cohort for comparison. Given the small sample size, analysis of other known clinical prognostic factors, such as FIGO stage and lymph node status, was not performed. Multivariate analysis was not performed because of the small number of patients included in this study. Univariate Cox proportional hazards and Kaplan-Meier/log-rank analyses was performed dichotomizing variables by the median (Table 3). Only the Pearson’s correlation coefficient significantly stratified patients for DFS by the log-rank test (*p* = 0.026), with a stronger correlation between SUV and ADC portending a worse prognosis (Table 3, Fig. 4). No variables reached significance on the univariate Cox analysis. No imaging metrics were significantly prognostic for overall survival by either Cox proportional hazards analysis or Kaplan-Meier/log-rank analysis (Additional file 1: Table S1).

**Table 3:** Univariate Cox proportional hazards analysis and log-rank analysis for DFS for the imaging metrics investigated. Variables were treated as categorical, dichotomized by the median for each imaging metric

Variable	Cox proportional hazards analysis	Log-rank
ADC_mean_	1.212 [0.270–5.439] *p* = 0.80	*p* = 0.80
ADC_T/M_	3.12 [0.60–16.11] *p* = 0.18	*p* = 0.16
SUV_max_	1.312 [0.292–5.885] *p* = 0.72	*p* = 0.72
MTV	3.637 [0.722–18.31] *p* = 0.12	*p* = 0.095
Pearson’s *r*	7.925 [0.934–67.23] *p* = 0.058	*p* = 0.026^†^

^†^*p* ≤ 0.05

## Discussion

[^18^F]FDG-PET is an established prognostic imaging marker in cervical cancer, and more recently diffusion-weighted MRI has also emerged as a potential imaging marker [3–8, 10, 12, 26]. The relationship between global tumor PET and diffusion MR imaging metrics, such as SUV_max_ and the ADC_min_, has previously been evaluated in cervical cancer, with data suggesting that these metrics are related [13, 17, 27]. The spatial relationship between ADC and SUV has qualitatively been described as well [21]. However, little is known about quantifying the spatial relationship between SUV and ADC, or the prognostic significance of the relationship between these metrics. Furthermore, there are limited data addressing the significance of histology and tumor grade on the relationship between these imaging metrics in cervical cancer [27].

With regard to global tumor metrics from [^18^F]FDG-PET and ADC images, our data showed a correlation between SUV_max_ and ADC_mean_, and SUV_max_ and ADC_T/M_ when all tumors were considered (Fig. 1). The same is true with SUV_mean_ and ADC_mean_ and ADC_T/M_ (Additional file 1: Figure S1). Results from prior studies have been heterogeneous, with some showing a relationship between SUV and ADCs, and others showing mixed result or no correlation between these values [13, 14, 16, 27]. Interestingly, in our patient cohort, we found a strong correlation between the SUV_max_ and the ADC_mean_ and ADC_T/M_, as opposed to the ADC_min_ which has previously been reported [13]. Normalizing tumor ADC to surrounding musculature has been reported for other disease sites in the pelvis [28], and may warrant further investigation for cervical cancer, as it may help minimize variability in ADCs between scans. In the case of our study, it may have helped offset differences in ADCs from different *b* values used in the scans included.

We also investigated how histology impacts global tumor metrics. In our patient cohort, there was no significant difference between SCCAs and AdenoCAs in the average values for the diffusion metrics (ADC_mean_, ADC_T/M,_ ADC_min_, and ADC_vol_) or the PET metrics investigated (SUV_max_, SUV_mean_, MTV, and TLG). With regard to the relationship between global tumor PET and ADC metrics, in our cohort, there was a significant inverse correlation between SUV_max_ and ADC_mean_ in AdenoCAs, and though there was an inverse correlation between these metrics in SCCAs, it did not meet the threshold for significance (Fig. 1c, d).

Prior works investigating how histology impacts PET and MRI metrics have yielded diverse results. Differences in [^18^F]FDG-PET imaging metrics between different cervical cancer histologies have previously been reported in a large series, which showed that on average, SCCAs have a greater SUV_max_ than non-SCCAs [22]. Similar findings have been reported for other cancer types (e.g., non-small cell lung cancer) [29, 30]. This was not seen in our small patient cohort. Prior studies have reported conflicting results regarding differences in ADC values between SCCAs and AdenoCAs, but the largest reported series supports that SCCAs have lower ADC values than AdenoCAs [14, 26, 31]. In our series, the average ADC_mean_ and the ADC_T/M_ are lower in SCCAs compared to AdenoCAs, though this did not reach statistical significance (Table 2). With regard to correlation between global tumor SUV and ADC metrics, a previous study has reported an inverse correlation between relative SUV_max_ (defined as SUV_max_/SUV_mean_) and a relative ADC_min_ (ADC_min_/ADC_mean_) in AdenoCAs and adenosquamos cancers, but not SCCAs [14, 32]. Similarly, our data show an inverse correlation between SUV_max_ and ADC_mean_ in AdenoCAs, but not SCCAs, though the strength of these relationships could change with a larger patient cohort.

Simultaneous acquisition of PET and MRI has the advantage of accurate image co-registration and has allowed us to compare SUV and ADC on a voxel level. In this study, we have performed this comparison in regions of restricted diffusion and high [^18^F]FDG avidity. To our knowledge, this is the first work investigating this relationship in primary cervical cancer tumors. Our data showed a consistent and significant inverse correlation between voxel SUV and ADC values in all SCCAs. However, only three of the AdenoCAs showed a statistically significant inverse correlation between voxel ADC and SUV, and three did not (Figs. 2 and 3). There was not a significant difference in the mean Pearson’s *r* of the SUV/ADC correlation between the SCCAs and the AdenoCAs. There was a significant difference between the voxel SUV/ADC correlation in poorly differentiated tumors compared to well/moderately differentiated tumors (Fig. 3d). It is important to note that 7/9 of the poorly differentiated tumors were SCCAs, and 4/6 of the well/moderately differentiated tumors were AdenoCAs. Given the overlap between grade and histology, the individual impacts of histology and tumor grade cannot be determined in this small patient cohort. The difference seen between these histologies and tumor grades may reflect some underlying physiologic or biologic difference in these tumors. It may reflect decreased cell density, and therefore less restricted diffusion, in the AdenoCAs and well/moderately differentiated tumors [26, 31]. This would be consistent with the higher ADC_mean_ and ADC_T/M_ values seen in AdenoCAs as well. However, different degrees of correlation between voxel SUV and ADCs are seen in both SCCAs and AdenoCAs and within the different tumor grades, and a more complete, and possibly alternative, underlying mechanism should be sought.

We also found a significant correlation between MTV and the voxel SUV/ADC relationship (Fig. 3e). This may imply that volume averaging effects or other volume-dependent effects impact the relationship between voxel SUV and ADC values. However, this may also be impacted by histology as the three tumors without an inverse correlation between voxel SUV and ADC were small, well/moderately differentiated AdenoCAs. There was not a significant correlation between MTV and the voxel SUV/ADC relationship in SCCAs, even though some of the SCCAs had small volumes (Fig. 3e).

Finally, we examined the prognostic potential of PET/MRI metrics as an exploratory analysis in our patient cohort. This analysis did not separate patients by histology. Only univariate analysis was performed given our small patient cohort. Interestingly, when the imaging metrics were stratified by their median, the Pearson correlation coefficient of the voxel ADC vs. SUV comparison was prognostic of DFS by Kaplan-Meier/log-rank analysis (Table 3, Fig. 4). That is, tumors with a stronger inverse correlation between voxel SUV and ADC had poorer disease outcomes. The prognostic significance of this marker was found retrospectively, and given the correlation between MTV and the voxel SUV/ADC relationship, MTV may be confounding the impact of the voxel SUV/ADC correlation on DFS. This underscores that while interesting, these results must be confirmed prospectively in a larger patient cohort.

The primary limitation of this study is the small sample size and its retrospective nature. This likely explains the variability seen between some of our results and the results of some previous studies, many of which also have small sample size. It is certainly possible that some of the relationships reported here between PET and ADC metrics may change or be refuted in a larger dataset. The small number of patients also limited our statistical analysis. In particular, we did not correct for multiple testing, nor did we perform a multivariate statistical analysis comparing imaging metrics to other known prognostic factors, such as FIGO stage, lymph node involvement, and change in tumor uptake on post-treatment [^18^F]FDG-PET. In addition, our patient cohort is heterogeneous, and includes patients treated with definitive chemoradiation and surgery, and cancers of a variety of grades, histologies, and stages. The scanning protocols in this patient cohort were also somewhat heterogeneous. Namely, a majority of patients received a PET/CT scan prior their PET/MR and thus had a delayed PET/MR acquisition relative to tracer injection. Finally, in this study, tumor volumes were defined manually on the ADC images to encompass the diffusion-restricted regions of tumor, and this approach could be susceptible to bias. The results reported here should therefore be viewed as hypothesis generating and must be confirmed and validated in a larger prospective patient cohort.

Nevertheless, the differences between histologies and tumor grades in the relationship of voxel [^18^F]FDG-PET SUV to ADC are interesting findings that warrant further investigation. Further work will need to validate the findings presented here and should also focus on defining the biologic and physiologic mechanisms underlying the relationship between SUV and ADC, for example, cellular density, proliferation, immunologic infiltrate, and mutational status.

## Conclusions

This work has demonstrated that, in our cervical cancer patient cohort, voxel values of ADC and SUV are inversely correlated in all SCCAs, but their relationship in adenocarcinomas is indeterminate. There is also a significant difference in the voxel ADC/SUV correlation between poorly and well/moderately differentiated tumors. Furthermore, we have shown that the correlation between voxel ADC and SUV values may have prognostic significance, though this may be confounded by the correlation between MTV and the voxel SUV/ADC relationship. These findings must be confirmed and validated in a larger patient cohort, and future work should also determine the biology underlying the relationship between imaging metrics reported here.

## Additional file

Additional file 1: **Figure S1.** Comparison of the global tumor metrics of SUV_mean_ and ADC_mean_ (a), SUV_mean_ and ADC_T/M_ (b), SUV_T/M_ and ADC_mean_ (c), and SUV_T/M_ and ADC_T/M_ (d) for all tumors. There was a significant inverse correlation between SUV_mean_ and ADC_mean_ and ADC_T/M_, but no significant correlation between SUV_T/M_ and either ADC_mean_ or ADC_T/M_. When SCCAs and AdenoCAs are considered separately, there is no significant correlation between SUV_mean_ and ADC_mean_ for either AdenoCAs (e) or SCCAs (f). **Table S1.** Univariate Cox proportional hazards analysis and log-rank analysis for OS for the imaging metrics investigated. Variables were treated as categorical, dichotomized by the median for each imaging metric. (DOCX 8404 kb)

## Abbreviations

[^18^F]FDG: 2-Deoxy-2-[^18^F]-fluoro-D-glucose
ADC: Apparent diffusion coefficient
ADC_mean_: Mean apparent diffusion coefficient
ADC_min_: Minimum apparent diffusion coefficient
ADC_T/M_: Mean tumor-to-muscle apparent diffusion coefficient ratio
ADC_vol_: Tumor volume defined on the ADC image
AdenoCA: Adenocarcinoma
MRI: Magnetic resonance imaging
MTV: Metabolic tumor volume
PET: Positron emission tomography
SCCA: Squamous cell carcinoma
SUV_max_: Maximum standardized uptake value
SUV_mean_: Mean standardized uptake value
SUV_T/M_: Mean tumor-to-muscle standardized uptake value
TLG: Total lesion glycolysis
VOI: Volume of interest
