# Supplementary material for: Spatial relationship of 2-deoxy-2-[18F]-fluoro-D-glucose positron emission tomography and magnetic resonance diffusion imaging metrics in cervical cancer
Source: EJNMMI Res. 2018 Jun 15;8:52. doi: 10.1186/s13550-018-0403-7 (PMC6003894; doi:10.1186/s13550-018-0403-7)
Supplement: Supplementary file 1 — Figure S1. Comparison of the global tumor metrics of SUVmean and ADCmean (a), SUVmean and ADCT/M (b), SUVT/M and ADCmean (c), and SUVT/M and ADCT/M (d) for all tumors. There was a significant inverse correlation between SUVmean and ADCmean and ADCT/M, but no significant correlation between SUVT/M and either ADCmean or ADCT/M. When SCCAs and AdenoCAs are considered separately, there is no significant correlation between SUVmean and ADCmean for either AdenoCAs (e) or SCCAs (f). Table S1. Univariate Cox proportional hazards analysis and log-rank analysis for OS for the imaging metrics investigated. Variables were treated as categorical, dichotomized by the median for each imaging metric. (DOCX 8404 kb) [file 13550_2018_403_MOESM1_ESM.docx]

**
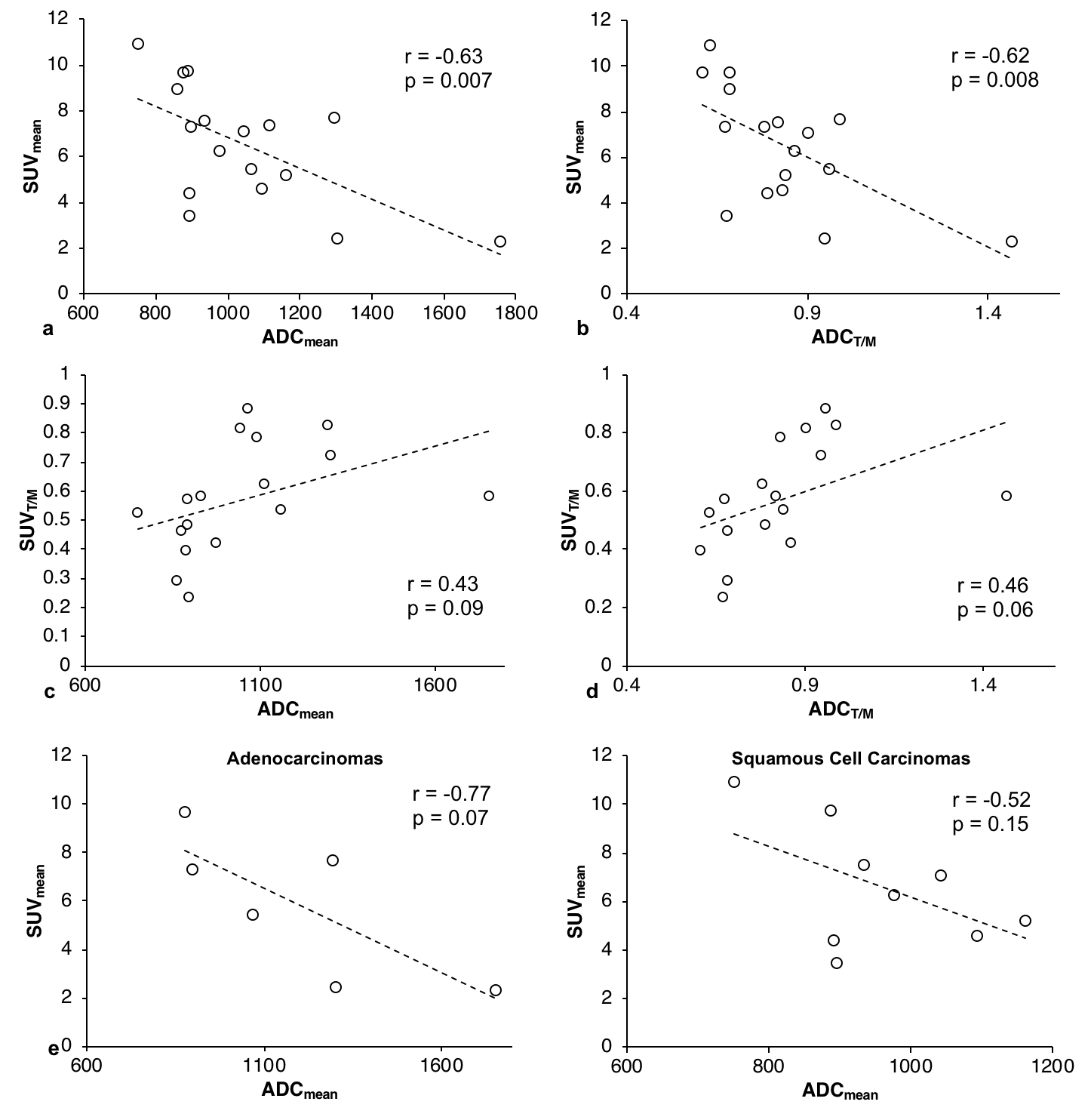
**

**Figure S1:** Comparison of the global tumor metrics of SUV_mean_ and ADC_mean_ (a), SUV_mean_ and ADC_T/M_ (b), SUV_T/M_ and ADC_mean_ (c), and SUV_T/M_ and ADC_T/M_ (d) for all tumors. There was a significant inverse correlation between SUV_mean_ and ADC_mean_ and ADC_T/M_, but no significant correlation between SUV_T/M_ and either ADC_mean_ or ADC_T/M_. When SCCAs and AdenoCAs are considered separately, there is no significant correlation between SUV_mean_ and ADC_mean_ for either AdenoCAs (e) or SCCAs (f).

**Table S1:** Univariate Cox proportional hazards analysis and log-rank analysis for OS for the imaging metrics investigated. Variables were treated as categorical, dichotomized by the median for each imaging metric.

| Variable | Cox Proportional Hazards Analysis | Log-Rank |
| --- | --- | --- |
| ADC_Mean_ | 0.318 [0.033-3.061] p = 0.32 | p = 0.30 |
| ADC_T/M_ | 1.278 [0.179-9.147] p = 0.81 | p = 0.81 |
| SUV_max_ | 1.125 [0.158-8.014] p = 0.91 | p = 0.91 |
| MTV | 1.125 [0.158-8.014] p = 0.35 | p = 0.91 |
| Pearson’s r | 3.415 [0.349-33.38] p = 0.29 | p = 0.26 |
|  | | |
